# Localized spatially nonlinear matter waves in atomic-molecular Bose-Einstein condensates with space-modulated nonlinearity

**DOI:** 10.1038/srep29566

**Published:** 2016-07-12

**Authors:** Yu-Qin Yao, Ji Li, Wei Han, Deng-Shan Wang, Wu-Ming Liu

**Affiliations:** 1 Department of Applied Mathematics, China Agricultural University, Beijing 100083, People’s Republic of China; 2 Beijing National Laboratory for Condensed Matter Physics, Institute of Physics, Chinese Academy of Sciences, Beijing 100190, People’s Republic of China; 3 Key Laboratory of Time and Frequency Primary Standards, National Time Service Center, Chinese Academy of Sciences, Xian 710600, People’s Republic of China; 4 School of Science, Beijing Information Science and Technology University, Beijing 100192, People’s Republic of China

## Abstract

The intrinsic nonlinearity is the most remarkable characteristic of the Bose-Einstein condensates (BECs) systems. Many studies have been done on atomic BECs with time- and space- modulated nonlinearities, while there is few work considering the atomic-molecular BECs with space-modulated nonlinearities. Here, we obtain two kinds of Jacobi elliptic solutions and a family of rational solutions of the atomic-molecular BECs with trapping potential and space-modulated nonlinearity and consider the effect of three-body interaction on the localized matter wave solutions. The topological properties of the localized nonlinear matter wave for no coupling are analysed: the parity of nonlinear matter wave functions depends only on the principal quantum number *n*, and the numbers of the density packets for each quantum state depend on both the principal quantum number *n* and the secondary quantum number *l*. When the coupling is not zero, the localized nonlinear matter waves given by the rational function, their topological properties are independent of the principal quantum number *n*, only depend on the secondary quantum number *l*. The Raman detuning and the chemical potential can change the number and the shape of the density packets. The stability of the Jacobi elliptic solutions depends on the principal quantum number *n*, while the stability of the rational solutions depends on the chemical potential and Raman detuning.

It is known that the precise control of untracold atomic systems have brought the realization of Bose-Einstein condensates (BECs) and Fermi gases. An important challenge is to produce and control more complicated molecular systems because of their potential applications for the tests of fundamental physics and for the drifts of fundamental constants. To date, several atomic-molecular conversion schemes have been provided^1^,^2^,^3^,^4^,^5^,^6^,^7^,^8^,^9^,^10^. Among them, Feshbach resonance^5^,^6^ and photoassociation^7^,^8^ are two main techniques to produce cole molecules from an atomic BECs. In real experiment, the cold molecules can be produced from a Fermi gas of atoms^11^,^12^ or an atomic BECs based on Feshbach resonance, Raman photoassociation or stimulated Raman adiabatic passage^13^,^14^,^15^. For example, a two-photon stimulated Raman transition in a ^87^*Rb* BECs has been used to produce ^87^*Rb*_2_ molecules in a single rotational-vibrational state^16^, where the input Raman laser pulse couples the molecular levels and reduces spontaneous emission. There is a nonlinear resonant transfer between atoms and molecules, as well as term proportional to the densities in the coupled atomic-molecular BECs. This type of soliton solutions have been studied in the nonlinear optics^17^,^18^ and in the problem of the self-localization of impurity atoms BECs^19^. The parametric solitons have been investigated in ref. 20. The coherent dynamics of this coupled atomic-molecular BECs have also been studied, which shows very rich behaviors, such as exact dark states solution^21^,^22^, crystallized and amorphous vortices^23^, Rabi oscillations^24^ and so on.

The intrinsic nonlinearity is the most remarkable characteristic of the BECs systems. In the past years, many interesting experiments, for example, the sonic-analogue of black holes, could be explored with spatial modulation of the interatomic interaction on short length scales. In refs 25 and 26, a promising technique (optical Feshbach resonance) is proposed to control the scattering length. With the development of this topic, a successful control of a magnetic Feshbach resonance of alkali-metal atoms was illustrated in ref. 27. In ref. 28, submicron control of the scattering length has been demonstrated by applying a pulsed optical standing wave to a BECs of ytterbium (^174^*Yb*) atoms. In recent research, the Nonlinear Schr*ö*dinger equation (NLSE) or the Gross-Pitaevskii equation (GPE) with spatially dependent cubic and quintic nonlinearities can be applied to the pulse propagation on optical fiber^29^, photonic crystals^30^, and the study of BECs^31^,^32^. The wide localized soliton solutions, the wide vector solutions, the dark soliton solutions and so on have been worked out^33^,^34^,^35^,^36^. The localized nonlinear waves in quasi-two-dimensional BECs with spatially modulated nonlinearity and in two-component BECs with time- and space- modulated nonlinearities are constructed^37^,^38^,^39^. However, there is few work considering the two-dimensional atomic-molecular BECs with space-modulated nonlinearities.

In this paper, we investigate the nonlinear matter waves in the two-dimensional atomic-molecular Bose-Einstein condensates with space-modulated nonlinearities, which can be described by the coupled GP equations with space-modulated nonlinearities. We work out three kinds of localized nonlinear wave solutions for both the attractive spatially inhomogeneous interactions and the repulsive ones by using the similarity transformation^40^. Our results show that the topological properties of the localized nonlinear matter waves given by the Jacobi elliptic function can be described by the principal quantum number *n* and the secondary quantum number *l*, while the topological properties of the localized nonlinear matter waves given by the rational function are independent of the principal quantum number *n*, only depend on the secondary quantum number *l*. The Jacobi elliptic solution is linearly stable only for the principal quantum number *n* = 1, while the stability of the rational form solutions depends on the chemical potential and Raman detuning.

## Results

### The coupled Gross-Pitaevskii equation with space-modulated nonlinearity

In real experiment, the coherent free-bound stimulated Raman transition can cause atomic BECs of ^87^*Rb* to produce a molecular BECs of ^87^*Rb*_2_. If the molecular spontaneous emission and the light shift effect can be ignored^41^,^42^, according to the mean field theory, the coupled atomic-molecular BECs^42^,^43^,^44^ with three-body interaction term can be written as


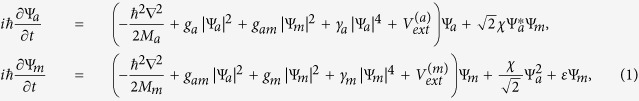


where 
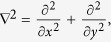
 Ψ_*i*_(*i* = *a*, *m*) denotes the macroscopic wave function of atomic condensate and molecular condensate respectively, 
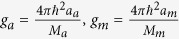
, 
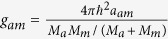
, *γ*_*a*_ and *γ*_*m*_ represent respectively the cubic and quintic nonlinearity the strengths of interaction, 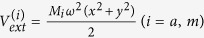 are the trapping potentials, *M*_*a*_(*M*_*m*_) is the mass of atomic (molecule), *χ* is the parametric coupling coefficient which describes the conversions of atoms into molecules due to stimulated Raman transitions. The parameter *ε* characterizes Raman detuning for a two photon resonance^16^,^41^,^45^. Integrating along the transverse coordinates, the above equations for the wave functions Ψ_*i*_(*i* = *a*, *m*) in dimensionless form can be written as the coupled GP equations


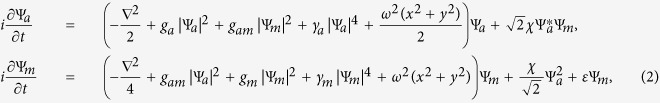


The unit of length, time and energy correspond to 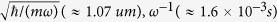 and *ħω*, respectively. In this paper, we use the parameters of atomic-molecular BECs of ^87^Rb system with *M*_*m*_ = 2 *M*_*a*_ = 2 *m* (*m* = 144.42 × 10^−27^ *Kg*), *g*_*m*_ = 2*g*_*a*_ (*a*_*a*_ = 101.8*a*_*B*_), where *a*_*B*_ is the Bohr radius.

Now we consider the spatially localized stationary solution 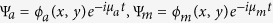 of ((2)) with *ϕ*_*i*_(*x*, *y*) (*i* = *a*, *m*) being a real function for *lim*_|*x*|,|*y*|→∞_*ϕ*_*i*_(*x*, *y*) = 0. This maps (2) into the following coupled equations


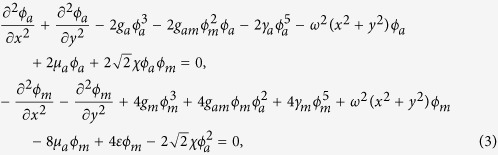


where *μ*_*a*_, *μ*_*m*_ are chemical potentials. In order to solve the above equations, we take the similarity transformation


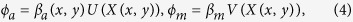


to transform (3) to the ordinary differential equations (ODEs)


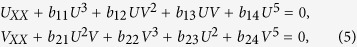


where *b*_*ij*_, *i* = 1, 2, *j* = 1, 2, 3 are constants. Substituting (4) into (3) and letting *U*(*X*), *V*(*X*) to satisfy (5), we obtain a set of partial differential equations (PDEs). Solving this set of PDEs, we have


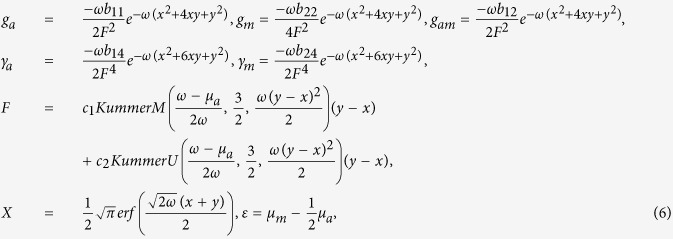


where 
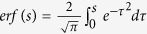
 is called an error function, 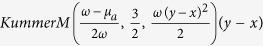 and 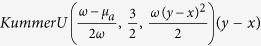 are solutions of the the ordinary differential equation


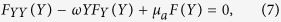


where *Y* = *y* − *x*. Specially, when 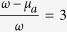, the KummerM function can be simplified as exponential function 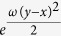. In this case, the interactions become as


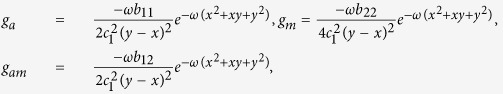


which is experimentally feasible due to the flexible and precise control of the scattering lengths achievable in BECs with magnetically tuning the Feshbach resonances^5^,^6^,^28^.

### Rational solution of the atomic-molecular BECs with three-body interaction

When the coupling *χ* = 0, (4) and (5) gives the rational formal solution of (2)


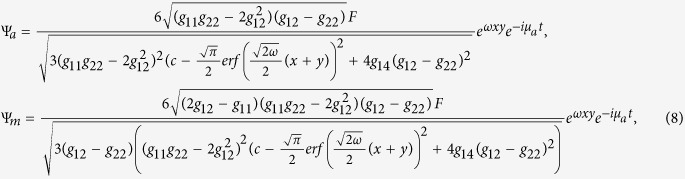


where *c* is arbitrary constant and *F* is given in (6).

**Figure 1:**
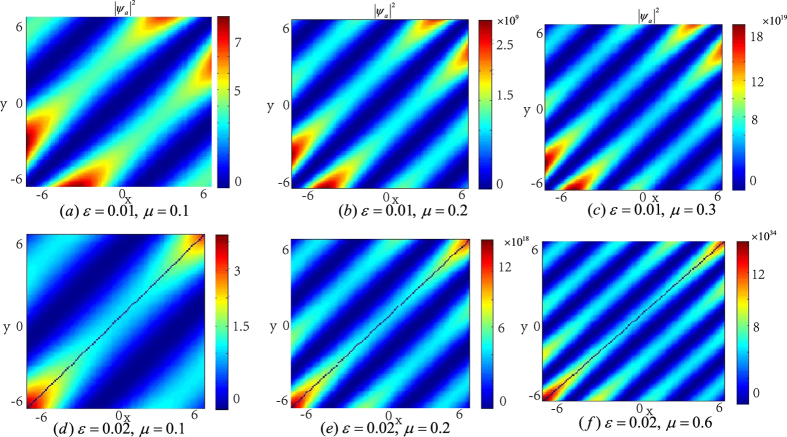
The density distributions |*ψ*_*a*_|^2^ of the atomic-molecular BEC with three-body interaction term as the function of *ε* and *μ*_*a*_ with *ω* = 0.02, *b*_11_ = 3, *b*_22_ = 12, *b*_12_ = 1. The energy packets are striped distribution. (**a–c**) show that the number of the energy stripes increases with chemical potential *μ*_*a*_ when *ε* is fixed. (**d–f**) illustrate that some zero points appear on the middle density stripe when the number of the density stripes is odd.

In order to investigate the topological properties of the exact spatially localized stationary solution (8), we plot their density distributions. In Fig. 1, it can be observed that the energy packets are striped distribution, and the number of the energy stripes increases with the chemical potential *μ*_*a*_ when *ε* is fixed. It can also be seen that some zero points appear on the middle density stripe along line *y* = *x* when the number of the density stripes is odd.

### Jacobi elliptic function solution

When the three-body effect is very weak and the coupling *χ* = 0, we have the following exact solutions of (5),


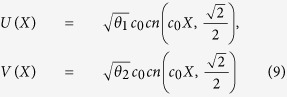


or


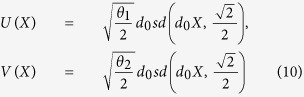


where *c*_0_, *d*_0_ are arbitrary constants, 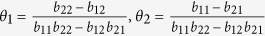 and *cn*, *sd* = *sn*/*dn* are Jacobi elliptic functions. When imposing the bounded condition *lim*_|*x*|,|*y*|→∞_*ϕ*_*i*_(*x*, *y*) = 0, we have 
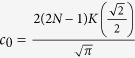
 and 
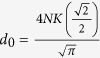
, where *N* is a natural number and 
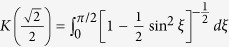
.

From Eqs (4), (6), (9) and (10), we obtain the Jacobi elliptic function solutions for the atomic-molecular BEC (2)


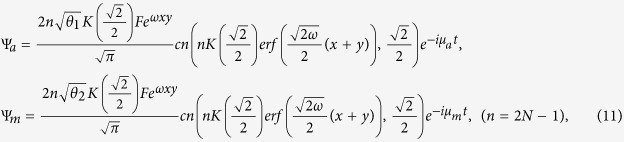


or


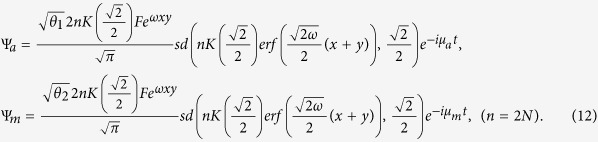


Here we discuss the existence regions of the spatially localized stationary solution (11) and (12) by assuming the two constraint conditions *θ*_1_ > 0 and *θ*_2_ > 0. We have the eight cases of parameters *b*_11_, *b*_12_ and *b*_22_. According to the real experiment, we consider the following two cases:

1. *b*_22_ < *b*_11_ < 0 and 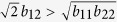.
2. *b*_22_ > *b*_11_ > 0 and 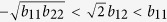.

These correspond to two cases of the intercomponent interaction parameters *g*_*a*_, *g*_*m*_ and *g*_*am*_:

- *g*_*m*_ > *g*_*a*_ > 0 and 
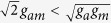
.
- *g*_*m*_ < *g*_*a*_ < 0 and 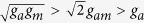.

These are the regions that the exact spatially localized stationary solutions (11) and (12) exist. Now we only consider case (b), which denotes two self-attractive atom-atom interactions, two self-attractive molecular-molecular interactions, and attractive and repulsive atomic-molecular interactions. The other cases can be analysed in the same way.

**Figure 2:**
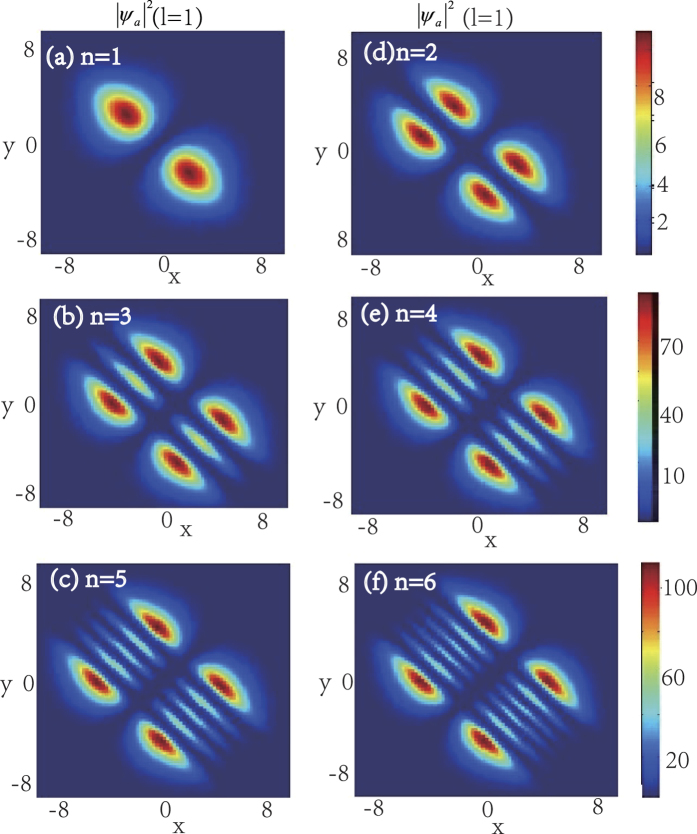
The density distributions |*ψ*_*a*_|^2^ of the atomic BEC as the function of the principal quantum number *n* when the secondary quantum number is fixed. The wave function *ψ*_*a*_ take the form in Eqs (11) and (12) with *b*_11_ = 3, *b*_22_ = 12, *b*_12_ = 1 and *ω* = 0.2. The number of density packets for each quantum states is equal to 2*n*. (**a–c**) show the density distributions of the odd parity wave functions (11) for *n* = 1, 3, 5, respectively. (**d–f**) illustrate the density distributions of the even parity wave functions (12) for *n* = 2, 4, 6, respectively. The solution displayed in figuer (**a**) is linear stable. The unit of the length is 1.07 *μm*.

**Figure 3:**
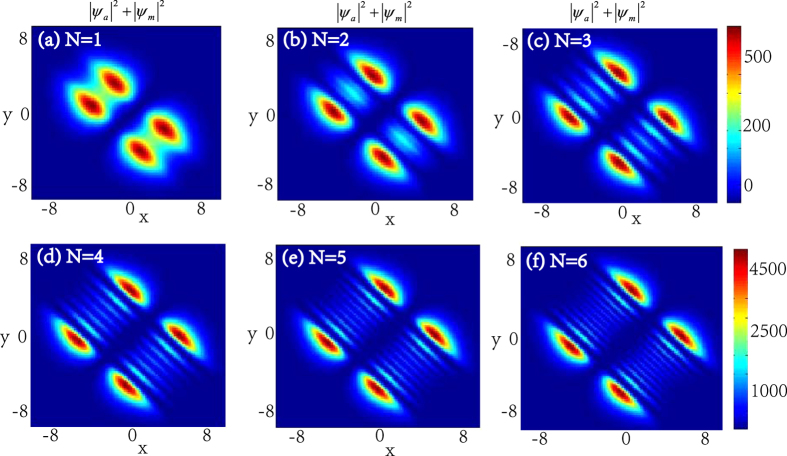
The density distributions |*ψ*_*a*_|^2^ + |*ψ*_*m*_|^2^ of the atomic-molecular pair as the function of *N* when the secondary quantum number *l* = 1. Here *b*_11_ = 3, *b*_22_ = 12, *b*_12_ = 1, and *ω* = 0.2. (**a**) shows the interaction of the atomic-molecular pair for *N* = 1. (**b**) shows the interaction of the middle atomic-molecular pair for *N* = 2, and it also displays that the interactions are weaker than the interaction in (**a**). (**a–f**) illustrate that the interaction becomes weaker with the increasing of *N*. The unit of the length is 1.07 *μm*.

In the following, we will see that the integer *n* and the number of the zero points of function *F* which equals to that of the KummerU and KummerM functions determine the topological properties of the atom and molecular packets, so we call *n* and *l* as the principal quantum number and the secondary quantum number, respectively. In order to investigate the topological properties of the exact spatially localized stationary solution (11) and (12), we plot their density distributions by manipulating the principal quantum number *n* when the secondary quantum number *l* is fixed. In Fig. 2, we analyse the atomic BEC when the secondary quantum number *l* is fixed and the principal quantum number *n* is modulated. It is easy to see that the number of density packets for each quantum states is equal to 2*n*. And the number of density packets on each quantum states increases two by two when the principal quantum number *n* increases. The properties of the molecular BEC are similar to that of the atomic BEC. In Fig. 3, we analyse the interactions of the atomic BEC and the molecular BEC when the secondary quantum number *l* is fixed. It is shown that the interaction is stronger when *N* = 1 and becomes weaker with the increasing of *N*.

**Figure 4:**
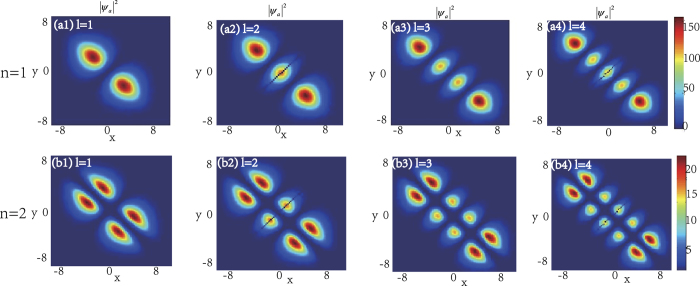
The density distributions |*ψ*_*a*_|^2^ of the atomic-molecular BEC as the function of the secondary quantum number *l*. Here *ω* = 0.2, *b*_11_ = 3, *b*_22_ = 12, *b*_12_ = 1. The number of the nodes for each quantum state equals to the secondary quantum number *l* and some zero points appear on the middle one when the number of the density packets is odd. (**a1–a4**) show the density distributions of the odd parity wave functions (11) for *n* = 1 and *l* = 1, 2, 3, 4, respectively. (**b1–b4**) show the density distributions of the even parity wave functions (12) for *n* = 2 and *l* = 1, 2, 3, 4, respectively. The solutions displayed in the first and third figures on the upper row are linear stable. The unit of the length is 1.07 *μm*.

**Figure 5:**
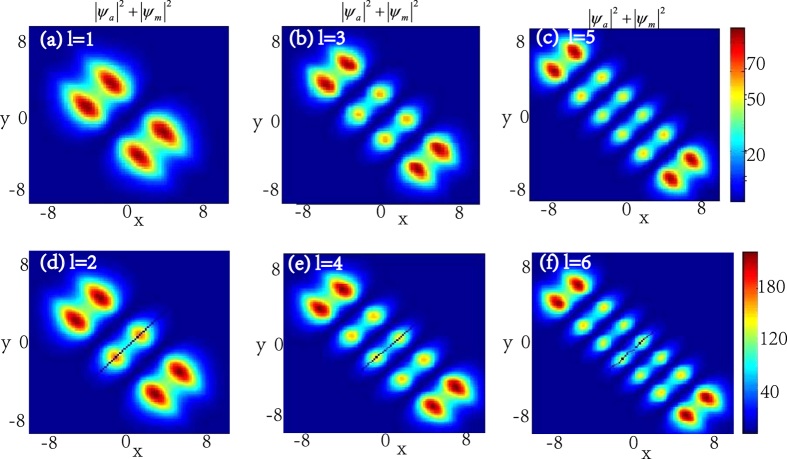
The density distributions |*ψ*_*a*_|^2^ + |*ψ*_*m*_|^2^ of the atomic-molecular pair as the function of the secondary quantum number *l* with *ω* = 0.2, *b*_11_ = 3, *b*_22_ = 12, *b*_12_ = 1. The number of the atomic-molecular pairs equals to *l* + 1. (**a–c**) show the density profiles of the atomic-molecular pair for *l* is odd. (**d–f**) show the density profiles of the atomic-molecular pair for *l* is even, and it also displays that some zero points appear on the middle one. The unit of the length is 1.07 *μm*.

When the principal quantum number *n* is fixed, we can adjust the secondary quantum number *l* to observe the properties of the atomic-molecular BEC. Figure 4 demonstrates the density distributions of atomic-molecular BEC for different secondary quantum number *l*. It is easy to find that the number of energy packets increases when *l* increases, and the number of the nodes for each quantum state equals to the secondary quantum number *l*. And some zero points appear on the middle density packets along the line *y* = *x* when the number of the secondary quantum number *l* is even. Figure 5 demonstrates the interaction of the atomic BEC and molecular BEC when the principal quantum number *n* is fixed. It is shown that the number of the atomic-molecular pair is the function of the secondary quantum number *l* and some zero points appear on the middle atomic-molecular pair along the line *y* = *x* when the number of the secondary quantum number *l* is even.

**Figure 6:**
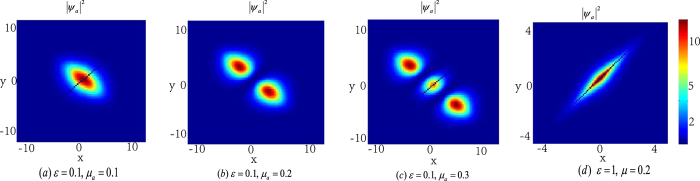
The effect of Raman detuning *ε* for the atomic-molecular BEC. (**a–c**) show that the number of the density packets increases with the chemical potential *μ*_*a*_ when *ε* < *μ*_*a*_. (**a,d**) reveal that the number of the density packets don’t depends on the chemical potential *μ*_*a*_ and there is only one density packet for each quantum state when *ε* ≥ *μ*_*a*_, it also show the value of *ε* − *μ*_*a*_ effects the shape of the density packet. The solution displayed in figure (**b**) is linear stable. The unit of the length is 1.07 *μm*.

Now we analyse the effect of Raman detuning *ε* for the atomic-molecular BEC. From Fig. 6, we can see that when *ε* < *μ*_*a*_ and *ε* is fixed, the number of the density packets increases one by one with the increasing of the chemical potential *μ*_*a*_. When *ε* ≥ *μ*_*a*_, there is only one density packets for each quantum states. The absolute of *ε* − *μ*_*a*_ affect the shape of the energy packets: when the absolute of *ε* − *μ*_*a*_ is small, the shape of the density packet is like circle, and when the absolute of *ε* − *μ*_*a*_ is larger, the shape of the density packet becomes narrow and long.

### Rational formal solution

When the three-body effect is very weak and the coupling *χ* ≠ 0, (4) and (5) also gives the rational formal solution of (2)


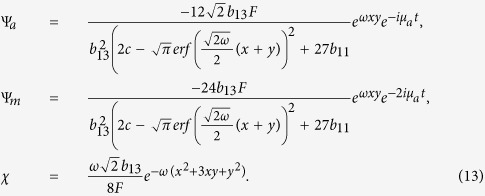


where *b*_13_ = *b*_23_, *b*_11_ > 0, *c* is arbitrary constant and *F* is given in (6).

**Figure 7:**
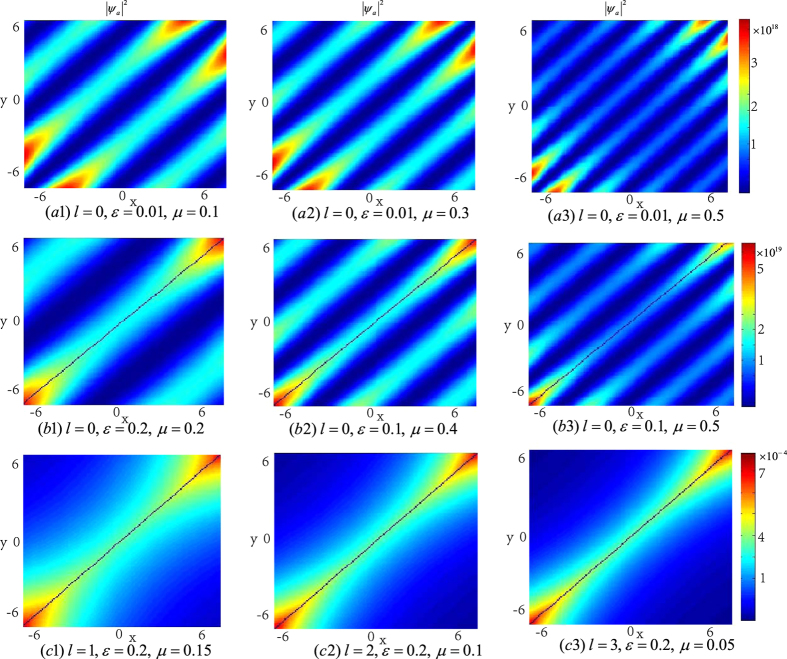
The density distributions |*ψ*_*a*_|^2^ of the atomic-molecular BEC as the function of *ε* and *μ*_*a*_ with *ω* = 0.02, *b*_11_ = 3, *b*_22_ = 12, *b*_12_ = 1. The energy packets are striped distribution. (**a1–b3**) show that the number of the energy stripes increases with chemical potential *μ*_*a*_ when the secondary quantum number *l* = 0. (**c1–c3**) show that there is only one density stripe when the secondary quantum number *l* ≠ 0. (**b1**,**c3**) illustrate that some zero points appear on the middle density stripe when the number of the density stripes is odd. The solutions displayed in figures (**a1,b1**) are linear stable.

In order to investigate the topological properties of the exact spatially localized stationary solution (13), we plot their density distributions by adjusting the secondary quantum number *l*. The secondary quantum number *l* is always zero for *ε* < *μ*_*a*_, and can be taken different values for *ε* ≥ *μ*_*a*_. In Fig. 7, it can be observed that the energy packets are striped distribution, and the number of the energy stripes increases with the chemical potential *μ*_*a*_ when the secondary quantum number *l* = 0 and *ε* is fixed. When the secondary quantum number *l* ≠ 0, there is only one energy stripe and the energy stripe becomes more narrower with the increasing of the secondary quantum number *l*. It can also be seen that some zero points appear on the middle density stripe along line *y* = *x* when the number of the density stripes is odd. Figures 1 and 7 show that the rational solution (8) and (13) have similar topological properties, which implies that three-body interaction doesn’t hinder the formation of the localized nonlinear matter wave solutions.

### Linear stability analysis

In the following, we analyse the linear stability of the solutions (11), (12) and (13) by using the linear stability analysis. A perturbed solution is constructed as^46^,^47^


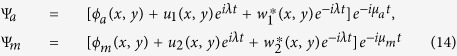


where |*u*_1_| ≪ 1, |*u*_2_| ≪ 1, |*w*_1_| ≪ 1, |*w*_2_| ≪ 1 are small perturbation. Substituting this perturbed solution into (2) and neglecting the higher-order terms in *u*_1_, *u*_2_, *w*_1_ and *w*_2_, we obtain the eigenvalue problem


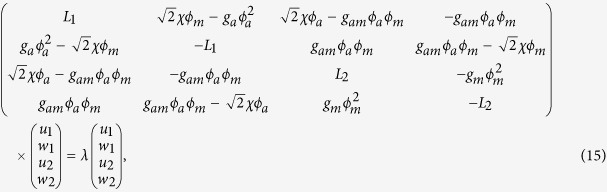


where


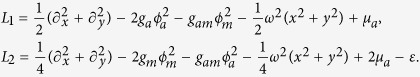


**Figure 8:**
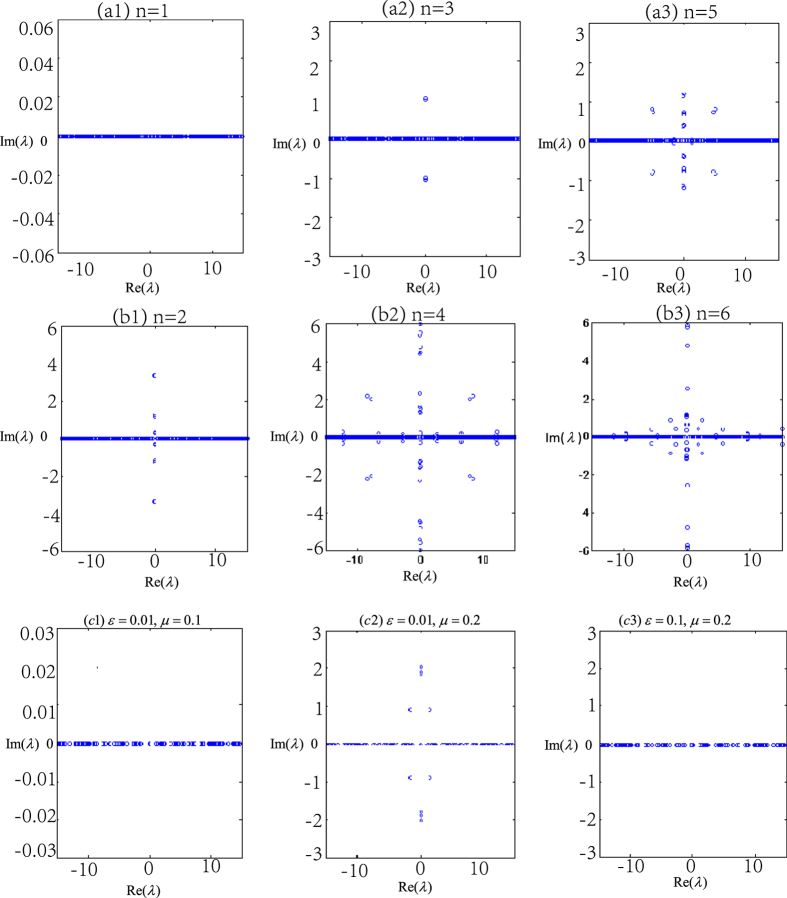
Linear stability. Eigenvalue for different principal quantum numbers *n* with parameters *b*_11_ = 3, *b*_22_ = 12, *b*_12_ = 1. (**a1–a3**) show that the exact solution (11) is linearly stable only for *n* = 1; (**b1–b3**) show that the exact solution (12) is linearly unstable for all *n*; (**c1–c3**) illustrate that the solution (13) are linearly stable in the two group parameters *ε* = 0.01, *μ*_*m*_ = 0.1 and *ε* = 0.1, *μ*_*m*_ = 0.2.

Numerical experiments show that the eigenvalue *λ* of the eigenvalue problem (15) is real for *n* = 1. This suggests that the localized nonlinear matter wave solution (11) is linearly stable for *n* = 1 and solution (12) is unstable. For the solution (13), it can be shown that the linear stability rests on the chemical potential *μ*_*a*_ and the Raman detuning *ε* (see Fig. 8).

## Discussion

In this paper, we focus on the analytic solutions of atomic-molecular BECs and the effects of the coupling *χ* and the Raman detuning *ε* on the atomic-molecular BECs. The system in this report is like the one in the ref. 43. Comparing to the atomic-molecular system given in the ref. 43, Gupta and Dastidar have proposed a more complicated model when they study the dynamics of atomic and molecular BECs of ^87^Rb in a spherically symmetric trap coupled by stimulated Raman photoassociation process in the ref. 42. In fact, the light shift effect in Gupta and Dastidar’s model almost has the same function as the Raman detuning term. So, it can be contributed to the Raman detuning term. Based on this reason, we don’t consider the light shift effect and take the form of the atomic-molecular BECs system as the form in ref. 43.

In the ref. 43, they show that the coherent coupling between atoms and molecules changes the situation crucially and it is sensitive to the presence of vortices. For example, when the coupling *χ* is zero, each of the atoms and molecular BECs wave function forms an independent triangular vortex lattice, and a nonzero coupling *χ* proposes more dramatic changes. Our results show that the coupling *χ* can change the topological structure of the localized nonlinear wave of the atomic- molecular BECs. In the case of *χ* = 0, Figs 1, 2, 3, 4 illustrate that the topological structures depend on the principal quantum number *n* and the secondary quantum number *l*, and each density packet is like a circle and oval. When *χ* ≠ 0, Fig. 6 display the density packets are striped distribution and their topological structures only reply on the secondary quantum number *l* and are independent on the principal quantum *n*.

In real experiment, spatial modulation of the interatomic interaction can be achieved. In the recent experiment^28^, the authors apply a pulsed optical standing wave to a BEC of ytterbium (^174^Yb) atoms and realize the submicron control of the scattering length. The experimental phenomena is well explained by the semi-classical theory of Bohn and Julienne^47^. In this paper, the interaction *g*_*a*_, *g*_*m*_, *g*_*am*_ and the coherent coupling *χ* all depend on the spatial variables. Under that conditions, the stable exact solutions can be worked out for the first time. The spatial modulation of the interaction can be realized by the above experiment, but there is no successful experiment for the spatial modulation of the coherent coupling. We hope that our research will stimulate the further research on the spatial modulation of the atomic- molecular BECs.

It is obvious that the Raman detuning term in the atomic- molecular BECs behaves just like the chemical potential to control the system’s energy. In this paper, the results imply that *μ*_*a*_ − *ε* not only changes the altitude of the wave packets, but also changes the topological structures of the nonlinear waves. When *μ*_*a*_ − *ε* ≥ 0, the number of the energy packets changes with the chemical potential *μ*_*a*_. When *μ*_*a*_ − *ε* < 0, there is only one energy packet for each quantum state.

In summary, we have worked out three kinds of localized nonlinear matter wave solutions of the two-dimensional atomic-molecular BECs with space-modulated nonlinearity and considered the effect of three-body interaction on the localized nonlinear matter wave solutions. Our results show that the matter wave functions given by elliptic function have even parity for the even principal quantum number and odd parity for the odd one, the number of density packets for each quantum state is twice of the principal quantum number *n*, and the number of density packets increases two by two with the principal quantum number *n*. The number of the nodes equals to the secondary quantum number *l*. For the nonlinear matter wave given by rational function, the number of the energy stripes increases with the chemical potential *μ*_*a*_ when the secondary quantum number *l* = 0 and *ε* is fixed. When the secondary quantum number *l* ≠ 0, there is only one energy stripe for each quantum state and the energy stripe becomes more narrower with the increasing of the secondary quantum number *l*. Odd (even) secondary quantum number *l* leads to even (odd) number of the energy packets (stripes). Some zero points appear on the middle energy packets (stripes) along line *y* = *x* for even secondary quantum number *l*. We also analyse the effect of Raman detuning *ε* for the atomic-molecular BECs. The value of *ε* − *μ*_*a*_ can change the number and shape of the energy packets (stripes). The stability of our solutions is analysed: the nonlinear matter wave solution (11) is linearly stable for the principal quantum number *n* = 1, the solution (12) is unstable, and the stability of the solution (13) rests on the chemical potential *μ*_*a*_ and the Raman detuning *ε*. Our results are significant to matter wave management in high-dimensional atomic-molecular BECs.

## Methods

We use the coupled Gross-Pitaevskii equation to describe the atomic-molecular BECs. Taking into account the term responsible for the creation of molecules^48^, the Hamiltonian is taken as


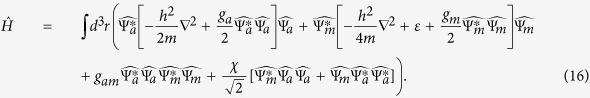


First, the coupled Gross-Pitaevskii equation is decomposed into two ODEs and a number of PDEs making use of the similarity transformation. Then we solve these ODEs and PDEs by using some solving techniques and some special functions, such as error function, KummerU function and Jacobi elliptic function. The final interaction parameters are altered to 
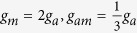
 and the chemical potential satisfies *μ*_*m*_ = 2*μ*_*a*_.

## Additional Information

**How to cite this article**: Yao, Y.-Q. *et al*. Localized spatially nonlinear matter waves in atomic-molecular Bose-Einstein condensates with space-modulated nonlinearity. *Sci. Rep.* **6**, 29566; doi: 10.1038/srep29566 (2016).
